# Isolated signals in BCL2, MYC, BCL6, and DDIT3 FISH: implications for genetic alterations and protein dysregulation

**DOI:** 10.3389/pore.2026.1612284

**Published:** 2026-02-11

**Authors:** Zongchen Wei, Qiuyue Chen, Zhenbo Feng, Fang Tang

**Affiliations:** 1 Department of Pathology, The 924th Hospital of the Chinese People’s Liberation Army Joint Logistic Support Force, Guilin, China; 2 Department of Pathology, The First Affiliated Hospital of Guangxi Medical University, Nanning, China

**Keywords:** break-apart probe, fluorescence *in situ* hybridization, gene rearrangement, isolated signal, lymphoma, myxoid liposarcoma

## Abstract

**Objectives:**

Fluorescence *in situ* hybridization (FISH) break-apart probes are widely employed to detect gene rearrangements in malignant tumors. Notwithstanding their utility, the complex genetic alterations in tumors frequently give rise to isolated signals, the mechanisms underlying which remain poorly understood. This study aimed to elucidate the genetic causes of isolated FISH signals in lymphoma and myxoid liposarcoma samples, providing a more accurate basis for interpreting FISH results.

**Methods:**

Six cases of lymphoma and myxoid liposarcoma, which showed isolated signals for *BCL2*, *MYC*, *BCL6*, or *DDIT3* in FISH detection, were carefully screened. Whole genome resequencing (WGR) was employed to analyze the genetic variations present in these samples. In addition, immunohistochemistry was used to assess the expression levels of the corresponding proteins in these samples.

**Results:**

WGR results revealed that all six cases with isolated signals harbored target gene translocations, with 5′and 3′probe-binding region deletions or inversions detected in *BCL2*, *MYC*, and *BCL6*, and in the 5′probe-binding region of *DDIT3*. Additionally, overexpression of the corresponding proteins was present in samples with isolated *BCL2*, *MYC*, and *BCL6* signals.

**Conclusion:**

Deletions or inversions in the probe-binding sequence regions may disrupt probe recognition and binding, leading to isolated FISH signals for *BCL2*, *MYC*, *BCL6*, and *DDIT3*. Notably, in cases with isolated *BCL2*, *MYC*, or *BCL6* signals, translocations involving these genes were associated with increased expression of their encoded proteins. These findings improve the understanding of FISH signal interpretation in tumor gene rearrangement detection and provide a valuable reference for clinical diagnosis.

## Introduction

Fluorescence *in situ* hybridization (FISH) break-apart probes are indispensable for detecting gene rearrangements in malignant cells. These probes utilize dual-color fluorophores to assess and visualize chromosomal integrity, facilitating the identification of structural aberrations such as translocations and inversions [1–4]. Typically, the probe hybridizes to the correct chromosomal location, generating the expected signals: intact loci yield fused signals, while translocations yield split signals [5–7]. However, tumors with complex genetic alterations frequently generate atypical signals, particularly isolated signals (characterized by the loss of signal from one probe) that challenge diagnostic paradigms [8–10]. Notably, in clinical practice, isolated signals are far rarer than classic split signals, which are commonly observed in translocation-positive malignancies. For classic split signals, the criterion for defining FISH positivity is well-established, usually requiring 15% or more of cells to display split signals [11–13]; in contrast, few reports specify the threshold proportion of cells with isolated signals that indicates FISH positivity, further complicating the standardized interpretation of such atypical signals [14]. Isolated signals are observed in only 0.1%–28.9% of all malignant cases undergoing break-apart FISH testing [8, 12, 13, 15–21]. Most large-cohort studies indicated rates below 10% [12, 19–21]. This rarity, together with the diagnostic ambiguity of isolated signals, further complicates accurate interpretation, as clinical laboratories often have limited experience with such infrequent signal patterns.

Isolated signals have been reported in break-apart probes for multiple genes, including *SS18* [5, 16, 17, 22], *BCL6* [18], *ALK* [12, 19, 20], *ROS1* [23, 24], *EWSR1* [13, 25], *DDIT3* [8], *FUS* [8, 26], *USP6* [8], *CBFB* [21], *MLL* [27] and *TFE3* [28], with varying interpretations across different studies. Notably, most existing hypotheses and inferences regarding the formation of these isolated signals have focused on the target genes themselves, primarily attributing their occurrence to deletions or translocations. For instance, isolated signals in the *SS18* break-apart probe, usually associated with loss of either the 5′or 3′signal, typically arise from specific unbalanced rearrangements [22], partial deletions of the *SS18* gene [17], or deletions of the *SS18-SSX* fusion gene [5]. Isolated 3′signals for *EWSR1* and *TFE3*, as well as isolated 5′signals for *CBFB,* are reported to arise from unbalanced rearrangements of the corresponding genes [13, 21, 28]. Isolated 5′signals in *ALK* may arise from deletions of the *ALK* 3′ region [19, 20]. Deletion of the 5′signal for *FUS* was thought to arise from supernumerary ring chromosomes [26]. For break-apart probes of *BCL6*, *ROS1*, *DDIT3* and *USP6*, isolated signals have occasionally been reported [8, 18, 23, 24], suggesting that further research is indicated. Although various hypotheses and inferences have been proposed regarding isolated signals, the exact mechanisms underlying them remain uncharacterized, with no consensus on their biological significance or clinical interpretation.

A recent large-cohort study demonstrated that break-apart probes of *MYC*, *BCL2*, *BCL6* can miss cryptic rearrangements due to small chromosomal insertions or inversions, yet their work did not address the distinct atypical pattern of isolated signals, whose genomic basis remains unclear [29].

In this study, six cases of lymphoma and myxoid liposarcoma with isolated FISH signals were investigated using genome resequencing to characterize the underlying genetic alterations. Additionally, the expression of the corresponding proteins was assessed. These investigations aimed to elucidate the mechanism of formation and diagnostic significance of isolated signals in FISH break-apart probe assays, which may ultimately aid in the development of evidence-based clinical guidelines.

## Materials and methods

### Case selection

This retrospective study enrolled 30 cases, including seven cases of follicular lymphoma (FL), five cases of Burkitt lymphoma (BL), 10 cases of diffuse large B-cell lymphoma (DLBCL), and eight cases of myxoid liposarcoma (MLPS). All of them were collected and analyzed from the pathological database and electronic medical records of the 924th Hospital of the Chinese People’s Liberation Army Joint Logistic Support Force between April 2017 and November 2023. Diagnostic confirmation of FL, BL, DLBCL, and MLPS was in accordance with morphological assessment, immunophenotype, and FISH screening. Any diagnostic discrepancies were resolved via a consensus between two senior pathologists. A consecutive sampling strategy was adopted in this study. All patients meeting the above diagnostic criteria and treated at the 924th Hospital of the Chinese People’s Liberation Army Joint Logistic Support Force during the study period were eligible for inclusion, with no exclusion based on patient characteristics (e.g., age, gender, Ann Arbor stage) or researcher subjective judgment. Cases were excluded if they met any of the following criteria: diagnostic uncertainty, insufficient sample quality for FISH and whole-genome resequencing, incomplete clinical data, or concurrent malignancies. Out of a total of 30 cases screened, six cases had isolated signals detected by BCL2, MYC, BCL6, or DDIT3 break-apart FISH probes. Four of the cases with classic split FISH signals were enrolled as controls. This study was approved by the Institutional Review Board/Ethics Committee of the 924th Hospital of the Chinese People’s Liberation Army Joint Logistic Support Force (approval number: GY-IRB-2023-009), and written informed consent was obtained from all participants.

### Fluorescence in situ hybridization

The 3-µm-thick formalin-fixed paraffin-embedded (FFPE) slides were deparaffinized, pretreated, and hybridized overnight with denatured probes for BCL2, MYC, BCL6, and DDIT3 (Guangzhou Lbp Medicine Science & Technology Co., Ltd.). The following morning, the slides were washed, stained with DAPI, mounted with a medium containing an antifade solution (Guangzhou Lbp Medicine Science & Technology Co., Ltd.), and examined using a Leica fluorescence microscope (Leica, Wetzlar, Germany). A classic split signal was defined as a fused signal with one red and one green signal (1F1R1G), whereas isolated signals contained either an isolated 5′signal or an isolated 3′signal. Two pathologists independently scored 100 non-overlapping nuclei per case, and discrepancies were resolved by a third reviewer.

### Whole genome resequencing (WGR)

DNA was extracted from seven 3-µm thick FFPE tissue sections using the QIAamp DNA FFPE Kit (Qiagen) per the manufacturer’s protocol. FFPE-associated artifact control: DNA integrity/purity via Agilent 2100 Bioanalyzer (Agilent DNA 1000 Kit; DNA Integrity Number ≥7.0, average fragment length ≥1000 bp) and spectrophotometry (A260/A280: 1.8–2.0, A260/A230 ≥ 1.5). The purified DNA was fragmented to approximately 300 bp using the Covaris S220 instrument. Libraries were then prepared with the VAHTS Universal Pro DNA Library Prep Kit (Vazyme). VAHTS DNA Clean Beads (Vazyme) were employed for sample cleanup and size selection, and VAHTS Dual UMI Adapters for Illumina (Vazyme) were used for ligation. Subsequently, the libraries were quantified using the Qubit 3.0 fluorometer, and their insert size distribution was examined using the Agilent 2100 Bioanalyzer with the Agilent DNA 1000 Kit (Agilent). Sequencing was performed using an Illumina NovaSeq 6000 (2 × 150 bp reads; NovaSeq 6000 S4 Reagent Kit v1.5, NovaSeq Xp 4-Lane Kit) with 0.25 nM phiX control. Post-sequencing quality control revealed: effective rate ≥85%, Q30 ≥ 80%, error rate ≤0.1%, GC content ∼40%–45% (consistent with human genome theoretical range), Ts/Tv ∼1.8–2.2 (typical for human genomes), InDel length primarily within ±30 bp; reads aligned to hg19 (Sentieon v202010-02) with average sequencing depth ≥20×, genome coverage ≥90%, PCR duplicate rate ≤25% (acceptable for tumor samples). Copy number variations (CNVs) were detected using ControlFREEC, and structural variations (SVs) were identified using LUMPY, both with uniform coverage. The variants were then annotated with ANNOVAR and visualized using IGV.

### Immunohistochemistry (IHC)

Slides were stained for IHC analysis using a Ventana BenchMark ULTRA (Ventana Medical System Inc., Tucson, AZ). The primary antibodies included BCL2, BCL6, and c-MYC (prediluted, ZSGB-BIO), and were visualized using enzyme peroxidase detection systems. Tonsil tissues served as positive controls. Two pathologists independently evaluated the slides after staining, and discrepancies were resolved by consensus review.

## Results

### Isolated signals detected in *BCL2*, *MYC*, *BCL6*, and *DDIT3* FISH break-apart probes

**FIGURE 1:**
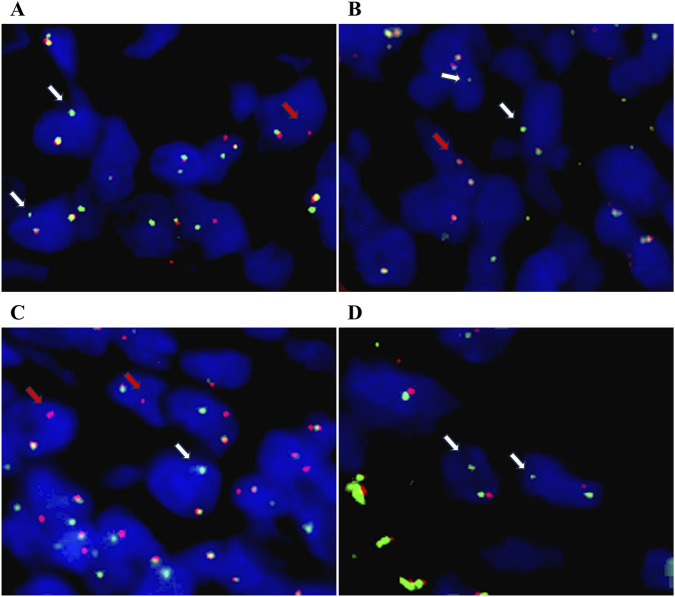
Atypical isolated signals detected by *BCL2, MYC, BCL6, and DDIT3* FISH break-apart probes. **(A)** Isolated 3'/5′signals in *BCL2* FISH break-apart probe (white arrow: isolated 3′signal; red arrow: isolated 5′signal). **(B)** Isolated 3'/5′signals in *MYC* FISH break-apart probe (white arrow: isolated 3′signal; red arrow: isolated 5′signal). **(C)** Isolated 3'/5′signals in *BCL6* FISH break-apart probe (white arrow: isolated 3′signal; red arrow: isolated 5′signal). **(D)** Isolated 3′signals in *DDIT3* FISH break-apart probe (white arrow). Original magnification: ×800.

In this study, a total of six cases with isolated signals (6/30, 20%), 18 cases with classic split signals (18/30, 60%), and six cases with negative signals (6/30, 20%) were assessed. Among the seven cases of FL, one showed distinct patterns of isolated 5'/3′BCL2 signals (1F1G, 1F1R, 2F1G) across different tumor cells, and six showed classic BCL2 split signals. Each cell with isolated signals displayed only one such pattern (no cell had multiple patterns simultaneously), and these isolated signals were present in 56% of tumor cells (Figure 1A; Table 1). For the five cases of BL, one showed isolated 5'/3′MYC signals (detected in 41% of tumor cells) and four showed classic MYC split signals. Each cell with isolated signals displayed only one pattern (either isolated 5′signals, including 1F1R, 1F2R1G, 2F1R, 2R1G, or isolated 3′signals, including 1F1G, 1F1R2G, 1F2G, 2F1G), with no cells exhibiting multiple patterns (Figure 1B; Table 1). In the 10 cases of DLBCL, BCL2, MYC, and BCL6 FISH break-apart probes were used separately. Among these, one showed isolated 5'/3′BCL6 signals, one showed classic BCL6 split signals, one showed classic MYC split signals, one showed concurrent classic BCL2 and BCL6 split signals, and six had only fused BCL2, MYC, or BCL6 signals. For the DLBCL case with isolated BCL6 signals, BCL6 FISH analysis revealed these signals in 39% of tumor cells; the signals were either 5'(1F1R, 2F1R, 2R1G, 1F2R, 1F2R1G) or 3′types (1F1G, 1F1R2G, 1F2G), with each cell harboring isolated signals displaying only one pattern (Figure 1C; Table 1). Among the eight cases of MLPS, three showed prominent DDIT3 telomeric signal deletions (isolated 3′signals), while five exhibited classic DDIT3 split signals. For the three cases of MLPS with DDIT3 telomeric signal deletions, atypical signals were observed in 54%–87% of tumor cells. Within each case, multiple distinct patterns were present across different tumor cells, though no single cell had more than one pattern. Specifically, Case 1 displayed 1F1G, 2F1G, 1F1R2G, and 1F3G patterns; Case 2 showed 2F2G, 1F2G, and 2F1G patterns; and Case 3 showed 2F1G and 1F1G patterns (Figure 1D; Table 1).

**TABLE 1:** Isolated signal types and patterns of *BCL2, MYC, BCL6,* and *DDIT3* FISH break-apart probes.

Gene	Isolated signal type	Signal pattern description	Associated disease	%
*BCL2*	Isolated 5'/3′signals	1F1G [26/100], 1F1R [25/100], 2F1G [5/100]	Follicular lymphoma	56
*MYC*	Isolated 5'/3′signals	1F1G [16/100], 1F1R [15/100], (1F1R2G, 1F2G, 2F1G, 1F2R1G, 2F1R, 2R1G) [10/100]	Burkitt lymphoma	41
*BCL6*	Isolated 5'/3′signals	1F1G [16/100], 1F1R [13/100], (1F1R2G, 1F2G, 2F1R, 2R1G, 1F2R, 1F2R1G) [10/100]	Diffuse large B-cell lymphoma	39
*DDIT3*	Isolated 3′signals	Case1: 1F1G [38/100], 2F1G [32/100], (1F1R2G, 1F3G) [8/100]	Myxoid liposarcoma	78
		Case2: 2F2G [35/100], 1F2G [27/100], 2F1G [25/100]		87
		Case3: 2F1G [31/100], 1F1G [23/100]		54

FISH, fluorescence *in situ* hybridization; %, percentage of tumor cells with isolated FISH signals.

### Quality control for FFPE samples in WGR

For the six FFPE tumor samples with isolated signals, post-sequencing quality control metrics revealed tumor purity from 80% to 90%, effective data rates ranging from 91.03% to 98.29%, Q30 ratios spanning from 93.24% to 93.97%, average error rates between 0.025% and 0.030%, GC contents from 41.55% to 44.33%, Ts/Tv ratios from 1.97 to 2.15, and InDel lengths predominantly within ±30 bp (Table 2). After alignment to the hg19 reference genome using Sentieon, the samples had average sequencing depths of 20.20× to 22.93×, genome coverage ranging from 91.59% to 92.24%, and PCR duplicate rates between 21.22% and 23.48% (Table 2). Moreover, CNVs, detected by ControlFREEC, and SVs, identified by LUMPY, showed uniform coverage, with no artifacts associated with FFPE interference with variant calling.

**TABLE 2:** Sequencing quality control metrics for the six FFPE samples with isolated signals in WGR.

Metrics	*BCL2*	*MYC*	*BCL6*	*DDIT3* case 1	*DDIT3* case 2	*DDIT3* case 3
Tumor purity (%)	80	90	80	80	80	80
Effective rate (%)	96.04	97.9	91.03	98.29	95.33	95.19
Q30 ratio (%)	93.62	93.24	93.49	93.97	93.59	93.31
Average error rate (%)	0.030	0.025	0.030	0.025	0.025	0.030
GC content (%)	41.98	41.78	44.33	41.55	42.56	43.12
Ts/Tv ratio	1.98	1.97	2.07	1.97	2.01	2.04
Average sequencing depth (×)	21.37	22.93	20.20	22.77	20.80	20.53
Genome coverage (%)	91.59	92.23	92.06	92.24	92.14	91.98
PCR duplicate rate (%)	22.93	22.42	21.22	23.18	24.78	24.04

### Translocations detected in isolated signals of *BCL2*, *MYC*, *BCL6,* and *DDIT3* break-apart probes in all six cases

**FIGURE 2:**
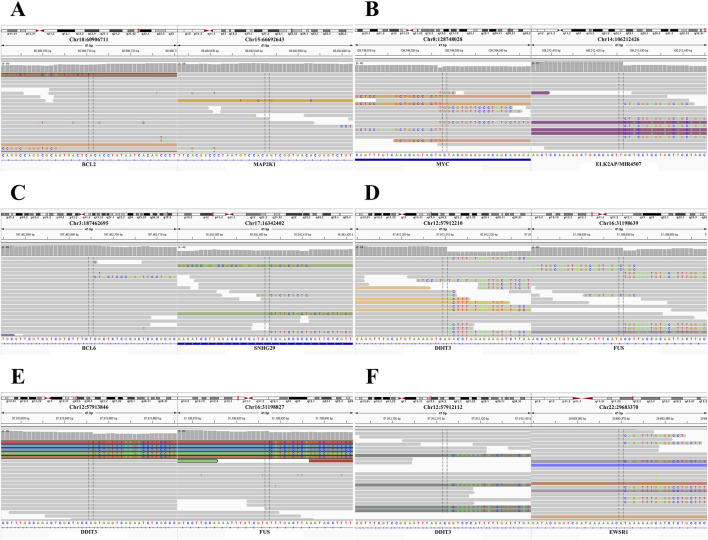
Translocations in isolated signals of *BCL2, MYC, BCL6,* and *DDIT3*. **(A)** *BCL2* and *MAP2K1* fusion in isolated 3'/5′*BCL2* signals, visualized by Integrative Genomics Viewer (IGV). **(B)** *MYC* and *ELK2AP/MIR4507* fusion in the isolated 3'/5′*MYC* signals, visualized by IGV. **(C)** *BCL6* and *SNHG29* fusion in isolated 3'/5′*BCL6* signals, IGV visualization. **(D,E)** *FUS-DDIT3* fusion detected in isolated 3′*DDIT3* signals, visualized by IGV. **(F)** *DDIT3* and *EWSR1* fusion in isolated 3′*DDIT3* signals, IGV visualization.

The WGR performed on the follicular lymphoma sample, which had isolated 5'/3′BCL2 signals, revealed a previously unreported fusion gene involving BCL2 and MAP2K1 (Figure 2A; Table 3). In the Burkitt lymphoma case, isolated 5'/3′MYC signals arose from a previously unreported intergenic fusion involving the MYC 5′untranslated region (UTR) and the ELK2AP/MIR4507 locus (Figure 2B; Table 3). In the DLBCL case, isolated 5'/3′BCL6 signals revealed a previously unreported fusion gene involving BCL6 and SNHG29 (Figure 2C; Table 3). In the MLPS cases, DDIT3 isolated 3′signals revealed classic and rare rearrangements: two cases were found to have canonical FUS-DDIT3 fusions (Figures 2D,E; Table 3), whereas the third case exhibited the rare EWSR1-DDIT3 fusion (Figure 2F; Table 3).

**TABLE 3:** Fusion genes and breakpoints of *BCL2, MYC, BCL6, and DDIT3* isolated signals.

Gene	Fusion partner	Breakpoint coordinates (hg19)	SU	PE	SR
*BCL2*	*MAP2K1*	chr18:60,906,711; chr15:66,692,643	5	1	4
*MYC*	*ELK2AP/MIR4507*	chr8:128,748,028; chr14:106,212,426	12	4	8
*BCL6*	*SNHG29*	chr3:187,462,695; chr17:16,342,402	7	2	5
*DDIT3*	*FUS*	chr12:57,912,210; chr16:31,198,639	18	4	14
*DDIT3*	*FUS*	chr12:57,913,846; chr16:31,198,827	17	6	11
*DDIT3*	*EWSR1*	chr12:57,912,112; chr22:29,683,370	14	6	8

SU, Supporting Unique; PE, paired-end; SR, split reads.

### Complex genetic alterations in probe-binding regions of isolated signals for *BCL2*, *MYC*, *BCL6* and *DDIT3*

**FIGURE 3:**
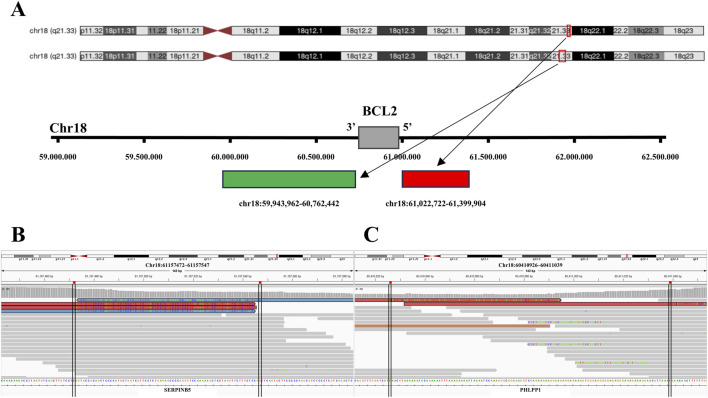
Deletions and inversions in the probe-binding regions of the tumor with *BCL2* isolated signals. **(A)** Tile coordinates of the *BCL2* FISH break-apart probe mapped onto the hg19 genome. The red region, indicated by the arrow, depicts the 5′probe-binding region mapping in the hg19 reference genome (Chr18:61022722 to Chr18:61399904). Similarly, the green region indicated, by the arrow, represents the 3′probe-binding region mapping in the hg19 reference genome (chr18:59943962 to Chr18:60762442). **(B)** In the sample with *BCL2* isolated 3'/5′signals, focal deletion (Chr18:61157472 to Chr18:61157547) is present in the 5′probe-binding region, visualized by IGV. **(C)** In the sample with *BCL2* 3'/5′isolated signals, micro-inversion (Chr18:60410926 to Chr18:60411039) is observed in the 3′probe-binding region, visualized by IGV.

In the follicular lymphoma case with isolated 5'/3′BCL2 signals, the 5′probe-binding region on chromosome 18q21.3 exhibited complex genetic alterations, including a focal deletion (Figures 3A,B; Table 4), two classes of inversions (Table 5), and multiple complex SVs, including inter- and intra-chromosomal translocations. Similarly, the 3′probe-binding region revealed alterations including an inversion (Figures 3A,C; Table 5), and diverse, complex SVs (inter- and intra-chromosomal translocations). By contrast, in the control case with classic BCL2 split signals, the 5′and 3′probe-binding regions on chromosome 18q21.3 exhibited only multiple complex SVs (inter- and intra-chromosomal translocations) without deletions or inversions.

**TABLE 4:** Deletions in probe-binding regions of *BCL2, MYC, BCL6, and DDIT3* isolated signals.

Gene	Probe region	Deletion coordinates	Size (bp)
*BCL2*	5′region	Chr18:61,157,472–61,157,547	75
*MYC*	5′region	chr8:127,864,438–128,294,955	430217
*MYC*	5′region	chr8:128,112,605–128,158,006	45401
*MYC*	5′region	chr8:128,235,243–128,370,620	135377
*MYC*	5′region	chr8:128,338,866–128,340,479	1613
*MYC*	5′region	chr8:128,611,296–128,611,392	96
*MYC*	3′region	chr8:129,441,264–129,694,921	253657
*MYC*	3′region	chr8:129,465,168–129,471,266	6098
*MYC*	3′region	chr8:129,575,496–129,575,531	35
*BCL6*	5′region	chr3:187,641,342–187,642,960	1618
*BCL6*	5′region	chr3:187,897,173–187,897,371	198
*BCL6*	5′region	chr3:188,032,773–188,032,848	75
*BCL6*	5′region	chr3:188,052,209–188,052,611	402
*BCL6*	5′region	chr3:188,110,867–188,111,239	372
*BCL6*	5′region	chr3:188,200,063–188,202,160	2097
*BCL6*	5′region	chr3:188,222,937–188,225,185	2248
*BCL6*	3′region	chr3:186,444,854–186,445,929	1075
*BCL6*	3′region	chr3:186,554,463–186,556,128	1665
*BCL6*	3′region	chr3:186,581,033–186,585,284	4251
*BCL6*	3′region	chr3:186,702,485–186,702,669	184
*BCL6*	3′region	chr3:186,795,865–186,796,188	323
*BCL6*	3′region	chr3:186,843,387–186,846,956	3569
*BCL6*	3′region	chr3:186,885,412–186,886,257	845
*BCL6*	3′region	chr3:186,969,267–187,034,809	65542
*BCL6*	3′region	chr3:187,018,626–187,423,899	405273
*BCL6*	3′region	chr3:187,065,789–187,065,938	149
*BCL6*	3′region	chr3:187,079,769–187,081,339	1570
*BCL6*	3′region	chr3:187,098,003–187,100,427	2424
*BCL6*	3′region	chr3:187,211,274–187,211,430	156
*BCL6*	3′region	chr3:187,276,760–187,276,884	124
*DDIT3*	5′region	chr12:58,435,905–58,436,066	161

**TABLE 5:** Inversions in probe-binding regions of *BCL2, MYC, BCL6, and DDIT3* isolated signals.

Gene	Probe region	Inversion coordinates	Type
*BCL2*	5′region	chr18:61,259,960–61,260,124	Multiple inversions
		chr18:61,347,431–61,347,823	
*BCL2*	3′region	chr18:60,410,926–60,411,039	Inversion
*MYC*	5′region	chr8:128,419,635–128,419,726	Micro-inversion
*MYC*	3′region	chr8:129,595,530–129,595,771	Inversion
*BCL6*	5′region	chr3:187,911,612–187,911,724	Multiple inversions
		chr3:188,065,571–188,065,774	
		chr3:188,205,663–188,205,984	
*BCL6*	3′region	chr3:186,505,961–186,506,093	Multiple inversions
		chr3:186,540,733–186,540,873	
		chr3:186,726,708–186,726,866	
		chr3:186,818,805–186,819,340	
		chr3:187,302,835–187,302,960	
		chr3:187,308,734–187,308,956	
		chr3:187,392,225–187,392,399	
		chr3:187,395,427–187,395,539	
*DDIT3*	5′region	chr12:58,096,050–58,096,229	Multiple inversions
		chr12:58,105,819–58,106,079	

**FIGURE 4:**
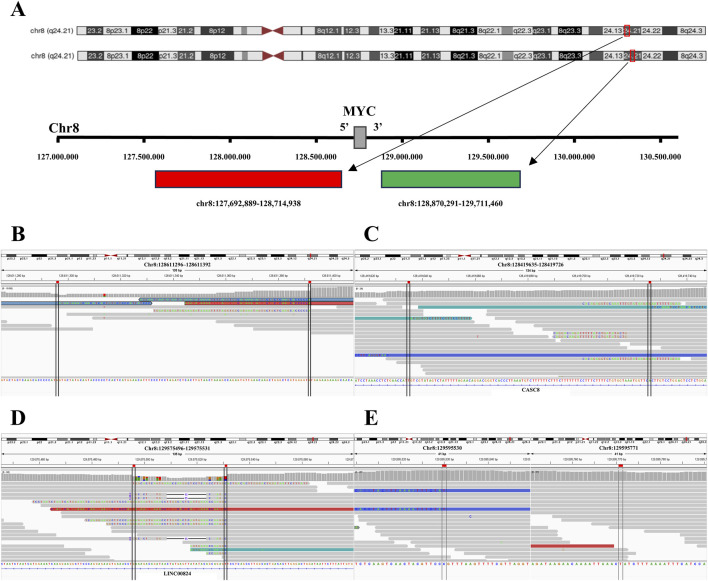
Deletions and inversions in the probe-binding regions of the tumor with *MYC* isolated signals. **(A)** Tile coordinates of the *MYC* FISH break-apart probe mapped onto the hg19 genome. The red region, indicated by the arrow, depicts the 5′probe-binding region mapping in the hg19 reference genome (Chr8:127692889 to Chr8:128714938). Similarly, the green region, indicated by the arrow, represents the 3′probe-binding region mapping in the hg19 reference genome (Chr8: 128870291 to Chr8:129711460). **(B)** In the tumor with *MYC* isolated 3'/5′signals, a focal deletion (Chr8:128611296 to Chr8:128611392) is observed in the 5′probe-binding region by IGV visualization. **(C)** In the tumor with *MYC* isolated 3'/5′signals, a micro-inversion (Chr8:128419635 to Chr8:128419726) is observed in the 5′probe-binding region by IGV visualization. **(D)** In the tumor with *MYC* isolated 3'/5′signals, a focal deletion (Chr8: 129575496 to Chr8:129575531) is observed in the 3′probe-binding region by IGV visualization. **(E)** In the tumor with *MYC* isolated 3'/5′signals, an inversion (Chr8:129595530 to Chr8:129595771) is observed in the 3′probe-binding region by IGV visualization.

In the Burkitt lymphoma case with isolated 5'/3′*MYC* signals, the 5′probe-binding region located at chromosome 8q24.21 exhibited five classes of deletions (Figures 4A,B; Table 4), a micro-inversion (Figures 4A,C; Table 5), and multiple SVs (inter- and intra-chromosomal translocations). The 3′probe-binding region revealed focal deletions (Figures 4A,D; Table 4), an inversion (Figures 4A,E; Table 5), and multiple SVs (inter- and intra-chromosomal translocations). In contrast, the control sample with classic *MYC* split signals demonstrated various SVs (inter- and intra-chromosomal translocations) without deletions or inversions identified at probe-binding regions.

**FIGURE 5:**
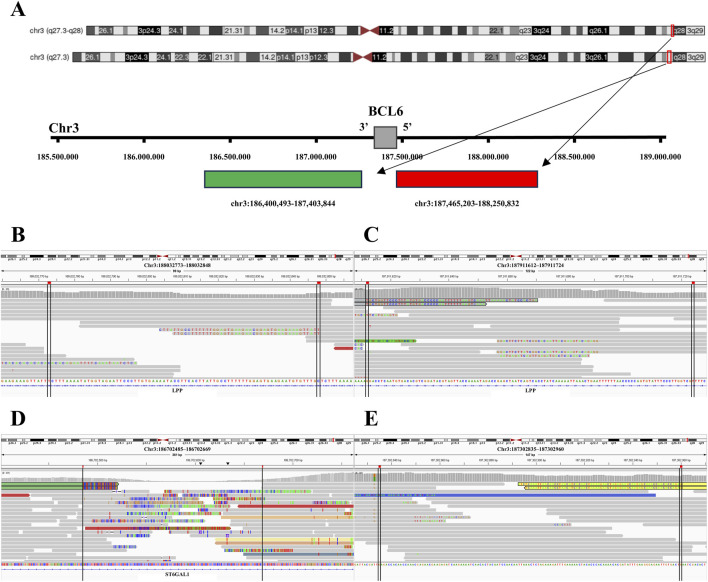
Deletions and inversions in the probe-binding regions of the tumor with *BCL6* isolated signals. **(A)** Tile coordinates of the *BCL6* FISH break-apart probe mapped onto the hg19 genome. The red region, indicated by the arrow, depicts the 5′probe-binding region mapping in the hg19 reference genome (Chr3:187465203 to Chr3:188250832). Similarly, the green region, indicated by the arrow, represents the 3′probe-binding region mapping in the hg19 reference genome (Chr3:186400493 to Chr3:187403844). **(B)** In the tumor with *BCL6* isolated 3'/5′signals, a focal deletion (Chr3:188032773 to Chr3:188032848) is observed in the 5′probe-binding region as visualized by IGV. **(C)** In the tumor with *BCL6* isolated 3'/5′signals, a micro-inversion (Chr3:187911612 to Chr3:187911724) is observed in the 5′probe-binding region as visualized by IGV. **(D)** In the tumor with *BCL6* isolated 3'/5′signals, a focal deletion (Chr3:186702485 to Chr3:186702669) is observed in the 3′probe-binding region by IGV visualization. **(E)** In the tumor with *BCL6* isolated 3'/5′signals, a micro-inversion (Chr3:187302835 to Chr3:187302960) is observed in the 3′probe-binding region by IGV visualization.

In the DLBCL case with isolated 5'/3′*BCL6* signals, the 5′probe-binding region at chromosome 3q27.3 demonstrated seven classes of deletions (Figures 5A,B; Table 4), three classes of inversions (Figures 5A,C; Table 5), and multiple SVs (inter- and intra-chromosomal translocations). The 3′probe-binding region showed fourteen classes of deletions (Figures 5A,D; Table 4), eight classes of inversions (Figures 5A,E; Table 5), and multiple SVs (inter- and intra-chromosomal translocations). For comparison, in the case with classic *BCL6* split signals, the 5′probe-binding region revealed limited deletions (chr3:186,581,033–186,585,284; chr3:186,826,665–186,826,969) and multiple SVs (inter- and intra-chromosomal translocations), but no inversions. The 3′probe-binding region also exhibited multiple SVs (inter- and intra-chromosomal translocations), without deletions or inversions.

**FIGURE 6:**
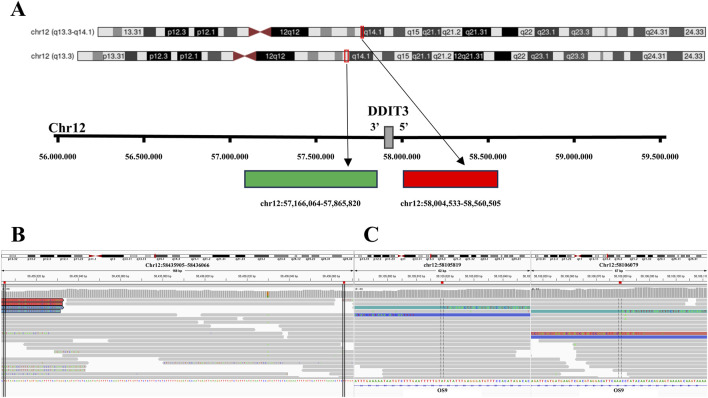
Deletions and inversions in the probe-binding regions of the tumors *DDIT3* isolated signals. **(A)** Tile coordinates of the *DDIT3* FISH break-apart probe mapped onto the hg19 genome. The red region, indicated by the arrow, depicts the 5′probe-binding region mapping in the hg19 reference genome (Chr12:58004533 to Chr12:58560505). Similarly, the green region, indicated by the arrow, represents the 3′probe-binding region mapping in the hg19 reference genome (Chr12:57166064 to Chr12:57865820). **(B)** In a tumor with *DDIT3* isolated 3′signals, a focal deletion (Chr12:58435905 to Chr12:58436066) is observed in the 5′probe-binding region as visualized by IGV. **(C)** In a tumor with *DDIT3* isolated 3′signal, a micro-inversion (Chr12:58105819 to Chr12:58106079) is observed in the 5′probe-binding region as visualized by IGV.

In the MLPS cases with *DDIT3* isolated 3′signals, one sample with *FUS-DDIT3* fusion showed a focal deletion at the 5′probe-binding region on chromosome 12q13.3 (Figures 6A,B; Table 4), and the other two cases displayed inversions (Figures 6A,C; Table 5). All three cases exhibited multiple SVs (inter- and intra-chromosomal translocations) at the 5′probe-binding region on chromosome 12q13.3. By contrast, a sample with classic *DDIT3* split signals showed only multiple SVs (inter- and intra-chromosomal translocations) at the 5′probe-binding region, without deletions or inversions.

### Overexpression of BCL2, c-MYC, and BCL6 in cases with isolated signals of those genes

**FIGURE 7:**
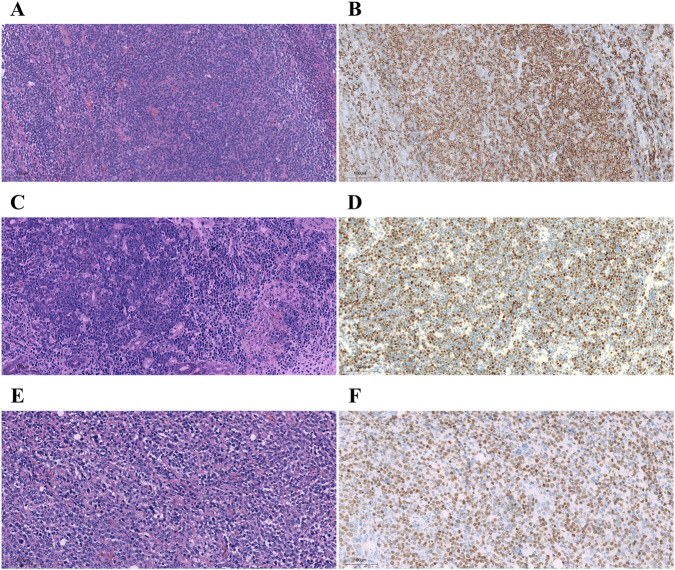
Expression of fusion proteins in *BCL2*, *MYC*, and *BCL6* isolated signal cases. In *BCL2* isolated 3'/5′signals, the follicular lymphoma case **(A)** showed diffuse BCL2 positivity **(B)**. In *MYC* isolated 3'/5′signals, the Burkitt lymphoma case **(C)** showed diffuse c-MYC positivity **(D)**. In *BCL6* isolated 3'/5′signals, the diffuse large B-cell lymphoma (DLBCL) case **(E)** showed diffuse BCL6 positivity **(F)**. Original magnification: ×200.

Protein expression was detected by immunohistochemical staining in cases with isolated signals for BCL2, MYC, and BCL6 (one case per gene). In the follicular lymphoma case with isolated 5'/3′BCL2 signals, BCL2 immunostaining demonstrated strong, diffuse membrane and cytoplasm expression in nearly 90% of tumor cells (Figures 7A,B), similar to that seen in samples without atypical signals. In the Burkitt lymphoma case with isolated 5'/3′MYC signals, c-MYC immunostaining revealed intense, diffuse tumor cell nuclear positivity in nearly 80% of tumor cells (Figures 7C,D). Similarly, in the DLBCL case with isolated 5'/3′BCL6 signals, BCL6 immunohistochemical staining showed strong, diffuse tumor cell nuclear expression in nearly 80% of tumor cells (Figures 7E,F).

## Discussion

The genetic heterogeneity of tumor cells often gives rise to atypical FISH signals, especially isolated signals, using break-apart probes to perform gene translocation analysis. In this study, we identified six cases exhibiting isolated FISH signals: three cases showed isolated 5'/3′signals for BCL2, MYC, or BCL6, while the remaining three cases displayed isolated 3′signals for DDIT3.

WGR performed on all six samples with isolated signals for *BCL2*, *MYC*, *BCL6*, or *DDIT3* revealed complex genomic rearrangements, with no case in this cohort showing isolated signals without concurrent genomic rearrangements. These changes included novel gene fusions (e.g., *MAP2K1*) involving *BCL2*, intergenic rearrangements (e.g., *ELK2AP/MIR4507*) affecting *MYC*, novel *BCL6* fusions (e.g., *SNHG29*), and rare *EWSR1*-*DDIT3* fusions (alongside the two classic *FUS-DDIT3* fusion). Distinct from previously reported fusion partners of *BCL2*, *MYC*, and *BCL6* [29–32], this is the first report, to our knowledge, of these novel partners for the three genes.

More significantly, complex genetic alterations, including deletions, inversions, and multiple SVs (inter- and intra-chromosomal translocations), were identified in the binding regions of FISH break-apart probes for the six cases with isolated signals by FISH analysis, with no deletions or inversions detected in the target genes (*BCL2, MYC, BCL6, DDIT3*) or their translocation partners [33]. For instance, deletions and inversions were detected in the 5′probe-binding region of BCL2 in our case, along with an inversion in its 3′probe-binding region. Deletions and inversions were also identified in both the 5′and 3′probe-binding regions of MYC and BCL6. Additionally, WGR analysis revealed complex genetic alterations in the 5′probe-binding regions of DDIT3, including large deletions, inversions, and multiple SVs. By contrast, in control tumor samples with classic split signals, the probe-binding regions of *BCL2*, *MYC* and *DDIT3* harbored only multiple SVs, whereas those of *BCL6* exhibited limited deletions alongside multiple SVs. This stark contrast suggests that extensive deletions or inversions in probe-binding regions are unique to tumors with isolated signal in the cases analyzed, and likely linked to their formation.

To interpret this distinction, we first wish to highlight the design principle of commercially available break-apart probes: probes targeting *BCL2, MYC, BCL6,* and *DDIT3* all adopt a dual-fluorophore strategy, labeling flanking sequences of the target gene (rather than the gene’s coding region itself) to assess chromosomal integrity (Figures 3A, 4A, 5A, 6A). Considering this mechanism and our WGR results, we propose that isolated signals arise due to complex genetic alterations in the probe-binding regions, rather than the target genes; deletions or inversions disrupt the recognition and binding of fluorophore-labeled probes, leading to the loss of one signal (red or green) and thus isolated signals. This mechanism is further supported by previous studies: Pacheco et al. [25] reported a deletion encompassing the *SMARCB1* locus on chromosome 22 in an atypical teratoid rhabdoid tumor case with isolated 3′*EWSR1* signals; Ordulu et al. [34] identified microdeletions in the telomeric and centromeric regions of 7p at the *JAZF1* locus in a low-grade endometrial stromal sarcoma case with 1F *JAZF1* signals; and Yang et al. [21] detected 16q inversions in eight acute myeloid leukemia cases with isolated 5′*CBFB* signals.

Zeng et al. [29] large-cohort study in DLBCL focused on *MYC*, *BCL2*, and *BCL6* and identified “FISH-cryptic rearrangements” (no observable signal abnormality, only detectable by NGS) caused by small insertions or inversions. In contrast, our study characterizes isolated signals as a distinct atypical pattern driven by deletions or inversions in probe-binding regions that highlights a unique genetic mechanism underlying this specific FISH signal anomaly.

Notably, in the cases analyzed, since all break-apart probes (targeting *BCL2*, *MYC*, *BCL6*, and *DDIT3*) share the core design principle of labeling target gene flanking regions, the identified cause of isolated signals was consistent across these probes (i.e., probe-binding region alterations rather than coding region-specific mechanisms). This observation provides a preliminary basis for exploring similar interpretations of isolated signals in other break-apart probe-targeted genes with analogous flanking sequence labeling principles, but generalizing interpretations of isolated signals across break-apart probe targeting genes with similar flanking sequence labeling principles requires further validation in larger, more homogeneous cohorts.

Immunohistochemical analysis revealed high expression of BCL2, c-MYC, and BCL6 in cases with isolated 5'/3′signals for the respective genes. WGR confirmed the presence of translocations involving these genes in all of the tumors with isolated signals, while no target gene amplification was detected. Tay et al. [17] detected the expression of SS18-SSX proteins in synovial sarcoma cases with isolated 5′*SS18* FISH signals, and next-generation sequencing results confirmed the occurrence of *SS18-SSX* fusions. However, in cases without fusion proteins, *SS18* translocation was not detected. Li et al. [19] detected ALK protein expression in non-small cell lung cancer cases with isolated 5′*ALK* signals and in those with isolated or attenuated 3′signals. Next-generation sequencing confirmed the occurrence of *ALK* fusions in these cases. Zeng et al. [29] revealed that all cases with FISH-cryptic *MYC*, *BCL2*, or *BCL6* rearrangements were positive for the corresponding proteins. In this study, the coexistence of high BCL2/c-MYC/BCL6 protein expression, confirmed gene translocations, and absence of target gene amplification strongly suggests that the observed upregulation of these proteins is likely driven by the translocations involving their respective genes, rather than by gene amplification events.

It is important to acknowledge this study’s limitations. First, only six isolated signal cases were analyzed, including heterogeneous malignancies (e.g., FL, BL, DLBCL, MLPS). The small sample size and inherent tumor type heterogeneity significantly weakens statistical power and restrict the generalizability of our findings. The proposed mechanism of isolated signal formation, therefore, may not apply to all tumor types, all break-apart probe-targeted genes, or larger, more homogeneous cohorts. Second, the single-center retrospective design of this study introduces additional potential biases. Reliance on archived samples meant that only specimens with sufficient tissue integrity and high-quality DNA extraction for WGR were included, while samples with severe DNA degradation, insufficient tissue volume, or poor preservation were excluded. This selection bias may have overrepresented cases with clear and detectable genetic alterations in probe-binding regions, potentially skewing the correlation between these alterations and isolated signal formation. Furthermore, tissue quality directly impacts WGR data accuracy: degraded DNA can lead to incomplete genomic coverage, missed detection of subtle deletions or inversions in probe-binding regions, and inaccurate identification of translocation breakpoints, all of which may compromise the reliability of our mechanistic inferences. Notably, the long-term stored archived samples also resulted in poor RNA quality, precluding RNA-based orthogonal confirmation of fusion transcripts. Additionally, the single-center setting limits the diversity of tumor subtypes and clinical backgrounds, further constraining the generalizability of our conclusions. As such, the present study should be explicitly considered a preliminary exploration of the genetic mechanism underlying isolated FISH signals. The conclusions drawn are tentative and require validation through future prospective studies featuring larger, well-stratified cohorts (with homogeneous tumor types and increased sample sizes) and longitudinal sampling to determine the broader applicability of the proposed mechanism. Additionally, no long-term follow-up data on treatment response and prognosis were collected, which precluded the assessment of the clinical implications of isolated FISH signals. Future studies with extended clinical follow-up are warranted to clarify the prognostic and therapeutic relevance of these atypical signals.

## Conclusion

Isolated signals detected by FISH break-apart probes for BCL2, MYC, BCL6, and DDIT3 may be attributed to deletions or inversions in the probe-binding sequences for these genes (not the target genes themselves). Notably, in cases with isolated BCL2, MYC, or BCL6 signals, our data showed an association between translocations involving these genes and increased expression of their encoded proteins.

## Data Availability

The original contributions presented in the study are included in the article/supplementary material, further inquiries can be directed to the corresponding author.

## References

[1] Menke JR Aypar U Bangs CD Cook SL Gupta S Hasserjian RP Performance of MYC, BCL2, and BCL6 break-apart FISH in small biopsies with large B-cell lymphoma: a retrospective cytopathology hematopathology interinstitutional consortium study. Front Oncol (2024) 14:1408238. 10.3389/fonc.2024.1408238 38903717 PMC11187077

[2] Lim SM Chang H Cha YJ Liang S Tai YC Li G Validation of ALK/ROS1 dual break apart FISH probe probe in non-small-cell lung cancer. Lung Cancer (2017) 111:79–83. 10.1016/j.lungcan.2017.07.016 28838404

[3] Aljerian K . FOXO1 and PAX5 rearrangement in alveolar rhabdomyosarcoma in Saudi pediatric patients. Fetal Pediatr Pathol (2023) 42(3):385–93. 10.1080/15513815.2022.2154134 36484735

[4] Bishop JA Koduru P Veremis BM Oliai BR Weinreb I Rooper LM SS18 break-apart fluorescence *in situ* hybridization is a practical and effective method for diagnosing microsecretory adenocarcinoma of salivary glands. Head Neck Pathol (2021) 15(3):723–6. 10.1007/s12105-020-01280-7 33394377 PMC8385014

[5] Zhong LL Huang GX Xian LY Wei ZC Tang ZP Chen QY Novel characteristics for immunophenotype, FISH pattern and molecular cytogenetics in synovial sarcoma. Sci Rep (2023) 13(1):7954. 10.1038/s41598-023-34983-2 37193761 PMC10188594

[6] Tobiášová K Barthová M Janáková Ľ Lešková K Farkašová A Loderer D Discordant ALK status in non-small cell lung carcinoma: a detailed reevaluation comparing IHC, FISH, and NGS analyses. Int J Mol Sci (2024) 25(15):8168. 10.3390/ijms25158168 39125737 PMC11312000

[7] Marino FZ Aquino G Brunelli M Scognamiglio G Pedron S Ronchi A High performance of multiplex fluorescence *in situ* hybridization to simultaneous detection of BCL2 and BCL6 rearrangements: useful application in the characterization of DLBCLs. Virchows Arch (2021) 479(3):565–73. 10.1007/s00428-021-03084-8 33768318 PMC8448700

[8] Papp G Mihály D Sápi Z . Unusual signal patterns of break-apart FISH probes used in the diagnosis of soft tissue sarcomas. Pathol Oncol Res (2017) 23(4):863–71. 10.1007/s12253-017-0200-z 28108880

[9] Murshed KA Abo Samra H Ammar A . Well-differentiated liposarcoma of the hypopharynx exhibiting myxoid liposarcoma-like morphology with MDM2 and DDIT3 Co-Amplification. Head Neck Pathol (2022) 16(1):288–93. 10.1007/s12105-021-01341-5 34089125 PMC9018935

[10] Ioannou M Perivoliotis K Zaharos NM Tsanakas A Tepetes K Koukoulis G . Alveolar rhabdomyosarcoma with unusual cytogenetic findings: one more case and review of the literature. Oxf Med Case Rep (2019) 2019(10):omz107. 10.1093/omcr/omz107 31798921 PMC6874863

[11] Tourneret A Alame M Rigau V Bauchet L Fabbro M De Oliveira L BCL2 and BCL6 atypical/unbalanced gene rearrangements in diffuse large B-cell lymphoma are indicators of an aggressive clinical course. J Clin Pathol (2021) 74(10):650–6. 10.1136/jclinpath-2020-206767 32912960

[12] Smuk G Pajor G Szuhai K Morreau H Kocsmár I Kocsmár É Attenuated isolated 3' signal: a highly challenging therapy relevant ALK FISH pattern in NSCLC. Lung Cancer (2020) 143:80–5. 10.1016/j.lungcan.2020.03.007 32272316

[13] Vargas AC Selinger CI Satgunaseelan L Cooper WA Gupta R Stalley P Atypical ewing sarcoma breakpoint region 1 fluorescence *in-situ* hybridization signal patterns in bone and soft tissue tumours: diagnostic experience with 135 cases. Histopathology (2016) 69(6):1000–11. 10.1111/his.13031 27385661

[14] Gagnon MF Penheiter AR Harris F Sadeghian D Johnson SH Karagouga G Unraveling the genomic underpinnings of unbalanced MYC break-apart FISH results using whole genome sequencing analysis. Blood Cancer J (2023) 13(1):190. 10.1038/s41408-023-00967-8 38114462 PMC10730864

[15] Amary MF Berisha F Bernardi Fdel C Herbert A James M Reis-Filho JS Detection of SS18-SSX fusion transcripts in formalin-fixed paraffin-embedded neoplasms: analysis of conventional RT-PCR, qRT-PCR and dual color FISH as diagnostic tools for synovial sarcoma. Mod Pathol (2007) 20(4):482–96. 10.1038/modpathol.3800761 17334349

[16] Yoshida A Arai Y Satomi K Kubo T Ryo E Matsushita Y Identification of novel SSX1 fusions in synovial sarcoma. Mod Pathol (2022) 35(2):228–39. 10.1038/s41379-021-00910-x 34504309

[17] Tay TKY Sukma NB Lim TH Kuick CH Goh JY Chang KTE . Correlating SS18-SSX immunohistochemistry (IHC) with SS18 fluorescent *in situ* hybridization (FISH) in synovial sarcomas: a study of 36 cases. Virchows Arch (2021) 479(4):785–93. 10.1007/s00428-021-03135-0 34091760

[18] Liew M Rowe LR Szankasi P Paxton CN Kelley T Toydemir RM Characterizing atypical BCL6 signal patterns detected by digital fluorescence *in situ* hybridization (FISH) analysis. Ann Lab Med (2018) 38(6):619–22. 10.3343/alm.2018.38.6.619 30027711 PMC6056388

[19] Li W Zhang J Guo L Chuai S Shan L Ying J . Combinational analysis of FISH and immunohistochemistry reveals rare genomic events in ALK fusion patterns in NSCLC that responds to crizotinib treatment. J Thorac Oncol (2017) 12(1):94–101. 10.1016/j.jtho.2016.08.145 27614248

[20] Guyard A Charpy C Théou-Anton N Cremades A Grassin F Bourgogne A Isolated 5' signals are an atypical pattern to be considered as positive for ALK rearrangement: a brief report of three cases and review of the literature. Transl Oncol (2019) 12(5):784–7. 10.1016/j.tranon.2019.02.015 30909092 PMC6434406

[21] Yang RK Toruner GA Wang W Fang H Issa GC Wang L CBFB break-apart FISH testing: an analysis of 1629 AML cases with a focus on atypical findings and their implications in clinical diagnosis and management. Cancers (Basel) (2021) 13(21):5354. 10.3390/cancers13215354 34771519 PMC8582369

[22] Jiang D Peng R Yan X Chen M Lan T Chen H Synovial sarcoma showing loss of a green signal in SS18 fluorescence *in situ* hybridization: a clinicopathological and molecular study of 12 cases. Virchows Arch (2017) 471(6):799–807. 10.1007/s00428-017-2211-2 28761985

[23] Mescam-Mancini L Lantuéjoul S Moro-Sibilot D Rouquette I Souquet PJ Audigier-Valette C On the relevance of a testing algorithm for the detection of ROS1-rearranged lung adenocarcinomas. Lung Cancer (2014) 83(2):168–73. 10.1016/j.lungcan.2013.11.019 24380695

[24] Yoshida A Kohno T Tsuta K Wakai S Arai Y Shimada Y ROS1-rearranged lung cancer: a clinicopathologic and molecular study of 15 surgical cases. Am J Surg Pathol (2013) 37(4):554–62. 10.1097/PAS.0b013e3182758fe6 23426121

[25] Pacheco MC Dolan M Bendel A . Ewing sarcoma and atypical teratoid rhabdoid tumor: a FISH and immunohistochemical comparison. Pediatr Dev Pathol (2017) 20(5):381–6. 10.1177/1093526617698599 28382842

[26] Bartuma H Möller E Collin A Domanski HA Von Steyern FV Mandahl N Fusion of the FUS and CREB3L2 genes in a supernumerary ring chromosome in low-grade fibromyxoid sarcoma. Cancer Genet Cytogenet (2010) 199(2):143–6. 10.1016/j.cancergencyto.2010.02.011 20471519

[27] Devi SG Goyal M Ramakrishna NV Murthy SS . CALLA negative precursor B lymphoblastic leukemia with MLL gene translocation and an unusual FISH signal pattern. Indian J Pathol Microbiol (2011) 54(1):176–9. 10.4103/0377-4929.77396 21393911

[28] Argani P Aulmann S Karanjawala Z Fraser RB Ladanyi M Rodriguez MM . Melanotic Xp11 translocation renal cancers: a distinctive neoplasm with overlapping features of PEComa, carcinoma, and melanoma. Am J Surg Pathol (2009) 33(4):609–19. 10.1097/PAS.0b013e31818fbdff 19065101

[29] Zeng Y Wei R Bao L Xue T Qin Y Ren M Characteristics and clinical value of MYC, BCL2, and BCL6 rearrangement detected by next-generation sequencing in DLBCL. Am J Surg Pathol (2024) 48(8):919–29. 10.1097/pas.0000000000002258 38937822 PMC11251499

[30] Yoon J Yun JW Jung CW Ju HY Koo HH Kim SH Molecular characteristics of terminal deoxynucleotidyl transferase negative precursor B-cell phenotype burkitt leukemia with IGH-MYC rearrangement. Genes Chromosomes Cancer (2020) 59(4):255–60. 10.1002/gcc.22825 31705772

[31] Ikoma H Miyaoka M Hiraiwa S Yukie Kikuti Y Shiraiwa S Hara R Clinicopathological analysis of follicular lymphoma with BCL2, BCL6, and MYC rearrangements. Pathol Int (2022) 72(6):321–31. 10.1111/pin.13223 35297566

[32] Gagnon MF Pearce KE Greipp PT Xu X Hoppman NL Ketterling RP MYC break-apart FISH probe set reveals frequent unbalanced patterns of uncertain significance when evaluating aggressive B-cell lymphoma. Blood Cancer J (2021) 11(11):184. 10.1038/s41408-021-00578-1 34819491 PMC8613271

[33] Zhang HG Zhang XY Zhang HY Tian T Xu SB Liu RZ . Balanced reciprocal translocation at amniocentesis: cytogenetic detection and implications for genetic counseling. Genet Mol Res (2016) 15(3). 10.4238/gmr.15038556 27706592

[34] Ordulu Z Avril S Nardi V Dias-Santagata D Oliva E . Low-grade endometrial stromal sarcoma with sex cord-like differentiation and PHF1-JAZF1 fusion with deletions: a diagnostic pitfall of JAZF1 FISH. Int J Gynecol Pathol (2022) 41(3):244–50. 10.1097/pgp.0000000000000795 34074959

